# Low implementation of Xpert MTB/RIF among HIV/TB co-infected adults in the International epidemiologic Databases to Evaluate AIDS (IeDEA) program

**DOI:** 10.1371/journal.pone.0171384

**Published:** 2017-02-09

**Authors:** Kate Clouse, Meridith Blevins, Mary Lou Lindegren, Marcel Yotebieng, Dung Thi Nguyen, Alfred Omondi, Denna Michael, Djimon Marcel Zannou, Gabriela Carriquiry, April Pettit, International Epidemiologic Databases to Evaluate AIDS (IeDEA) collaboration

**Affiliations:** 1 Vanderbilt Institute for Global Health, Nashville, Tennessee, United States of America; 2 Vanderbilt University Medical Center, Nashville, Tennessee, United States of America; 3 Vanderbilt Tuberculosis Center, Nashville, Tennessee, United States of American; 4 Department of Biostatistics, Vanderbilt University Medical Center, Nashville, Tennessee, United States of America; 5 College of Public Health, The Ohio State University, Columbus, Ohio, United States of America; 6 National Hospital of Tropical Diseases, Hanoi, Vietnam; 7 Academic Model Providing Access To Healthcare (AMPATH), Eldoret, Kenya; 8 National Institute for Medical Research (NIMR), Mwanza, Tanzania; 9 Faculté des Sciences de la Santé, Université d'Abomey-Calavi, Cotonou, Bénin; 10 Instituto de Medicina Tropical Alexander von Humboldt, Lima, Peru; McGill University, CANADA

## Abstract

**Objective:**

Xpert MTB/RIF is recommended by the World Health Organization (WHO) as the initial tuberculosis (TB) diagnostic test in individuals suspected of HIV-associated TB. We sought to evaluate field implementation of Xpert among a cohort of HIV/TB co-infected individuals, including availability, utilization and outcomes.

**Design:**

Observational cohort study (patient-level data) and cross-sectional study (site-level Xpert availability data).

**Methods:**

Data were collected at 30 participating International epidemiologic Databases to Evaluate AIDS (IeDEA) sites in 18 countries from January 2012-January 2016. All patients were HIV-infected and diagnosed with TB, either bacteriologically or clinically, and followed until a determination of TB treatment outcome. We used multivariable modified Poisson regression to estimate adjusted relative risk (RR) and 95% confidence intervals for unfavorable TB treatment outcomes.

**Results:**

Most sites (63%) had access to Xpert, either in the clinic (13%), in the same facility (20%) or offsite (30%). Among 2722 HIV/TB patients included, median age was 35.4 years and 41% were female; BMI and CD4 count were low. Overall, most patients (76%) received at least one TB test; 45% were positive. Only 4% of all patients were tested using Xpert: 64% were Xpert-positive, 13% showed rifampicin (RIF) resistance and 30% were extrapulmonary (EPTB) or both pulmonary-EPTB. Treatment outcomes were mostly favorable (77%) and we found little association between Xpert use and an unfavorable TB treatment outcome (RR 1.25, 95%CI: 0.83, 1.90).

**Conclusions:**

In this cohort, Xpert utilization was low even though the majority of sites had access to the test. Our findings show the need for expanded implementation and further research exploring barriers to use in low-resource settings.

## Introduction

The World Health Organization (WHO) estimates that effective diagnosis and treatment of tuberculosis (TB) saved approximately 49 million lives between 2000 and 2015.[1] However, despite these gains and notable global efforts like the Millennium Development Goals[2] and the End TB Strategy,[3] TB remains an enormous threat to global health. In 2015, 1.4 million people were estimated to have died from TB, of whom about one-fourth were co-infected with HIV.[1] WHO further estimates that over 41% of new cases in 2015 were undiagnosed or unreported.[1]

In the past decade, TB diagnostics received a significant advancement with the introduction of Xpert® MTB/RIF (Cepheid, Sunnyvale, CA, USA).[4] Xpert MTB/RIF (Xpert) is a polymerase-chain reaction (PCR) assay that simultaneously detects *Mycobacterium tuberculosis* and rifampicin resistance–a marker for multi-drug resistance[5]–and provides results in approximately two hours.[6] A number of clinical trials demonstrated improved sensitivity of Xpert over the conventional first-line diagnostic in resource-limited settings, smear microscopy, particularly among HIV-positive individuals.[7–10] Additionally, the reduced turnaround time for TB diagnostic results offered the potential for use of Xpert at the point of care.[4,11,12] In December 2010, WHO endorsed Xpert as the initial diagnostic test in individuals suspected of multi-drug resistant TB (MDR-TB) or HIV-associated TB,[13] and updated this policy in 2014 to expand the indication to the testing of children with HIV or suspected MDR-TB, as well as individuals suspected of having extrapulmonary TB (EPTB).[14]

Following the WHO guidelines, worldwide procurement expanded rapidly. As of the end of 2014, Xpert was reportedly available in 116 countries, with China and South Africa the largest procurers of test modules.[15] While the availability of Xpert has quickly spread, only a few studies have evaluated field implementation of Xpert. Noted operational and logistical challenges to the systematic implementation of Xpert in resource-limited settings include cartridge stock-outs, electricity interruptions, temperature control and increased human resources requirements.[11,16–18] Also, countries may delay their adoption of the WHO guidelines, or adapt them for their own context; for example, some countries recommend Xpert testing following a negative smear result.[19] This was also the case in the Democratic Republic of Congo, until a field evaluation demonstrated such an algorithm, combined with lower retention along the testing cascade, resulted in very few patients effectively being tested.[20]

The International epidemiologic Databases to Evaluate AIDS (IeDEA) network is an international research consortium funded in 2005 by the National Institutes of Health to provide a rich resource for globally diverse HIV/AIDS data (www.iedea.org). A 2012 survey of 47 sites in 26 countries within the IeDEA collaboration found that Xpert was available in nearly half of the sites (48.9%).[21] Although Xpert was available, that did not necessarily mean the test was being used for patient care. In order to evaluate the field implementation of Xpert, we expanded on this earlier IeDEA site-level study to collect patient-level data exploring the use of Xpert in IeDEA sites and association of Xpert use with TB outcomes.

## Methods

### Patient population

**Fig 1:**
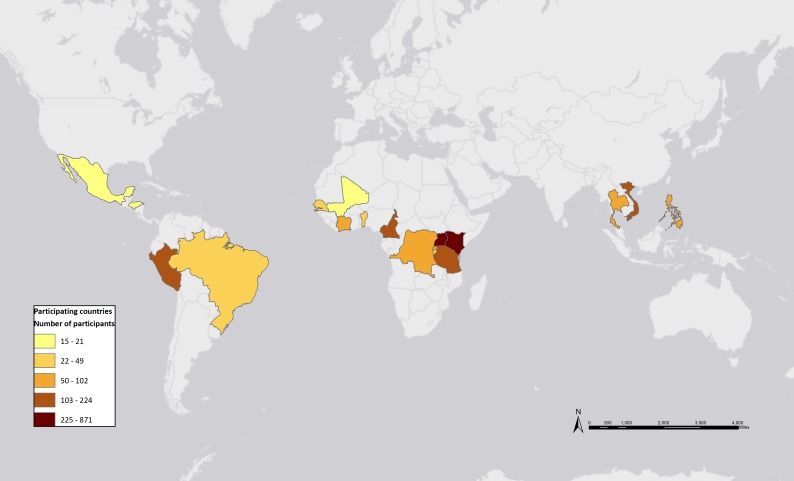
Participating countries (n = 18) and number of patients included by each. Map created in July 2016 by Kate Clouse using ArcMap GIS 10.3.1 (Esri, Redlands, CA).

We conducted an observational cohort study among HIV-infected adults enrolled in HIV care at participating IeDEA sites and diagnosed with tuberculosis during 2012–2014 (n = 2722). Forty adult IeDEA sites were invited to participate; thirty sites in 18 countries participated (Fig 1) and were a mix of hospital and primary-level facilities. All patients identified as TB/HIV co-infected and ≥16 years old were included. Patients were excluded if TB was initially suspected, but an alternative diagnosis was established and anti-tuberculosis therapy was discontinued. Recurrent cases were excluded (n = 41), so a patient would not contribute more than one TB case.

### Ethics statement

Independent Ethics Committee (IEC) or Institutional Review Board (IRB) approval for this study was obtained by each of the local IeDEA sites as well as from the Vanderbilt University IRB and included: Indiana University IRB, Comite de Etica do Instituto Nacional de Infectologia Evandro Chagas-Fiocruz (Brazil), Comité de Éticas en Investigación Biomédica (CEIB) of the Unidad de Investigación Científica (UIC) (Honduras), Comité de Ética en Investigación del Instituto Nacional de Ciencias Médicas y Nutrición, Salvador Zubirán (Mexico), Comité Institucional de Ética para Humanos (CIEH) and Comité Institucional de Ética en Investigación del Hospital Cayetano Heredia (CIEI) (Peru), Comité national d’éthique pour la recherche en santé (CNERS) (Benin), Comité National d'Ethique et de la Recherche (Côte d'Ivoire), Comité National d'Ethique pour la santé et les Sciences de la vie (Mali), Comité National d'Ethique pour la Recherche en Santé (Senegal), Moi University/Moi Teaching and Referral Hospital Institutional Research and Ethics Review Committee (Kenya), The United Republic of Tanzania National Institute for Medical Research Coordinating Committee (Tanzania-Tumbi and Kisesa), Mbarara University of Science and Technology Institutional Research Ethics Committee (Uganda), Comité National d’Ethique (Burundi), Cameroon National Ethical Committee of Research for Human Health (Cameroon), Comite D’ethique of the University of Kinshasa School of Public Health (République Démocratique du Congo), Rwanda National Ethics Committee (Rwanda), Research Institute for Tropical Medicine Department of Health (Philippines), Committee on Human Rights Related to Research Involving Human Subjects Faculty of Medicine Ramathibodi Hospital, Mahidol University (Thailand), Institutional Review Board Faculty of Medicine, Chulalongkorn University (Thailand, HIV-NAT), Ministry of Health, Hanoi School of Public Health Institutional Review Board (Bach Mai Hospital, Vietnam)*, The Ethical Review Board for Biomedical Research of National Hospital of Tropical Diseases (Vietnam). Written informed consent was waived by all committees except for the Research Institute for Tropical Medicine Department of Health (Philippines), Institutional Review Board Faculty of Medicine, Chulalongkorn University (Thailand, HIV-NAT), and Ministry of Health, Hanoi School of Public Health Institutional Review Board (Bach Mai Hospital, Vietnam). These three sites enrolled only participants >18 years of age, so a separate consent procedure for minors was not required.

### Site-level xpert availability data

Availability of Xpert was recorded during the site assessment survey in March-June 2012, and defined as a) available on site, b) available off site or c) not available.[21] Data were collected one time per facility by local site staff. Other data collected included type of HIV and TB services provided in-house and outside the facility. One site in the present study did not participate in the 2012 survey; it was noted that Xpert MTB/RIF became available at the site in 2012 at the time of the previous survey.

### Patient-level HIV and TB data

Patient-level demographic data as well as data on TB treatment, TB outcomes, and laboratory data were collected retrospectively using an electronic case report form developed in Research Electronic Data Capture (REDCap).[22] Electronic case report forms were completed by a local IeDEA site investigator for TB cases diagnosed at the site from January 2012-December 2014 following electronic and/or hard copy medical record review at local HIV and/or TB treatment clinical sites. Data entry took place from January 2012- January 2016. Patient-level data on antiretroviral therapy (ART) use was requested from the IeDEA regional data managers for all patients with an electronic case report form in REDCap.

### Study definitions

TB testing refers to smear microscopy, culture, Xpert MTB/RIF or other nucleic acid amplification test (NAAT). TB diagnoses were classified as either bacteriologically-confirmed or clinically-diagnosed cases as defined by WHO.[23] Bacteriologically-confirmed disease was defined as a positive culture, Xpert MTB/RIF or other NAAT, or positive acid-fast bacillus (AFB) on smear microscopy. Clinically-confirmed cases were defined by signs and symptoms consistent with TB as well as a clinical decision to give a patient a full course of anti-TB treatment, as determined by local investigators within the context of no TB diagnostic tests or a negative TB test result. Chest x-ray information was not collected and was not included in definitions of disease. The date of TB diagnosis was defined as the date of the first positive microbiologic test or clinical diagnosis of TB, whichever came first. The CD4+ lymphocyte count at TB diagnosis was defined as the nearest available value 180 days before or up to 30 days after the date of TB diagnosis. Xpert MTB/RIF testing was defined for each subject as not performed, performed, or unknown. Xpert MTB/RIF results were noted to be positive, negative, or invalid.

Patients were followed until TB treatment outcomes were determined as defined by WHO: cure, completion, failure, death, default and transfer. Favorable outcomes included a) cure and b) completion of treatment. Unfavorable outcomes included a) death during TB treatment, b) failure and c) default.

### Statistical analysis

We present counts and proportions for categorical variables and medians and interquartile ranges (IQR) for continuous variables. Poisson regression models with robust error variances were used to estimate relative risk (RR) and 95% confidence intervals (CI) for unfavorable TB treatment outcomes.[24] We excluded 220 patients (8%) from the regression analysis due to inconclusive results (25%), missing results (23%) or facility transfer (52%). Covariates identified *a priori* included age, sex, body mass index (BMI), CD4+ cell count at TB diagnosis, Xpert receipt, ART status at TB diagnosis, and region (to account for region-level heterogeneity). If there was evidence of non-linearity, then continuous variables were modeled using a restricted cubic spline with 4 knots. Multiple imputation with predictive mean matching was used to account for missing values of covariates and to prevent case-wise deletion of missing data.[25] A secondary analysis allowed for heterogeneity across regions using a meta-analysis approach to combine estimates across five regional models (S1 Appendix).[26] Statistical analyses were performed using R version 3.2.5 (www.r-project.org) and analysis scripts are available online (http://biostat.mc.vanderbilt.edu/ArchivedAnalyses).

## Results

Characteristics of the 2722 patients at the time of TB diagnosis are shown in Table 1. Median age was 35.4 years (IQR: 29.8–42.8) and 41% were female. Median BMI (19 kg/m^2^ [IQR: 17–21]) and CD4 count (117 cells/μl [IQR: 41–253]) were low. ART data were not available for (25%); of those with non-missing data, 1354 (66%) were not on ART at the time of TB diagnosis. Over half (56%) of the patients included in the study were from East Africa, with the rest of the patients split between the other study regions.

**Table 1:** Patient characteristics at time of TB diagnosis (n = 2722).

Age at TB diagnosis, *median (IQR)*	35.4 (29.8–42.8)
Female, *n(%)*	1111 (41%)
CD4 count, *median (IQR)*	117 (41–253)
Missing, *n(%)*	416 (15%)
BMI, *median (IQR)*	19 (17–21)
Missing, *n(%)*	597 (22%)
ART status at TB diagnosis, *n(%)*	
No ART	1354 (50%)
On ART	700 (26%)
6+ months at TB diagnosis	374 (14%)
< 6 months at TB diagnosis	326 (12%)
Missing ART status	668 (25%)
Region, *n(%)*	
Asia-Pacific	330 (12%)
Caribbean, Central and South America	309 (11%)
Central Africa	397 (15%)
East Africa	1522 (56%)
Western Africa	164 (6%)
Year of TB diagnosis, n(%)	
2012	1230 (45%)
2013	1023 (38%)
2014	469 (17%)

Table 2 shows Xpert availability at the participating sites at the time of surveying in 2012. Most sites (63%) had access to Xpert, either in the clinic (13%), in the same facility (20%) or offsite (30%). Xpert use was highest (13%) among patients at sites offering Xpert in the same facility.

**Table 2:** Xpert availability at participating sites.

Xpert availability	Participating Sites* (n = 30)	Patient Xpert receipt+ (n = 2722)
In this clinic	4 (13%)	28/754 (4%)
In the same health facility (but not at this clinic)	6 (20%)	44/326 (13%)
Only offsite (at distance)	9 (30%)	35/1153 (3%)
Not available	10 (33%)	11/453 (2%)
Availability unknown	1 (3%)	0/36 (0%)

*Site-level data on Xpert availability were obtained from a site assessment conducted in 2012.

+ Patient-level data on Xpert receipt were obtained from the REDCap database including TB cases diagnosed at the participating sites from January 2012-December 2014. The 11 patients noted as receiving an Xpert test in a site where Xpert was not available may be due to receiving an Xpert test at a non-study site or may reflect an increase in procurement in Xpert from the time of the initial site assessment in 2012.

TB testing utilization and outcomes are shown in Table 3. Overall, 2070 patients (76%) received at least one TB test, with a total of 2555 TB tests conducted, allowing for different test types. AFB smear was the most utilized test (n = 2025, 79%) followed by culture (n = 333, 13%), Xpert (n = 118, 5%) and other NAAT (n = 79, 3%). Nearly half (45%) of the 2070 patients tested were positive on at least one test. Most (81%) of the cases testing TB-positive were diagnosed as pulmonary TB, with 11% extrapulmonary TB (EPTB) and 8% both. TB treatment outcomes were missing for 8% of patients. Of those with data, the majority (77%) of patients overall (including those not tested) had a favorable TB treatment outcome; this did not differ by those with a positive TB test result (81%) compared to those with a negative result (80%).

**Table 3:** TB testing utilization and outcomes among 2722 adult patients.

	n (%)
TB test utilization (n = 2722)	
Received at least one TB test	2070 (76%)
Received no TB test	650 (24%)
Missing	2 (<1%)
Type of TB test performed (n = 2555)*	
AFB smear	2025 (79%)
Culture	333 (13%)
Xpert	118 (5%)
Other NAAT	79 (3%)
Among TB tested (n = 2070), TB test results	
At least 1 positive result	931 (45%)
Negative	1139 (55%)
Among test positives (n = 931), Site of disease	
Pulmonary	750 (81%)
Extrapulmonary	99 (11%)
Both	79 (8%)
Unknown	3 (< 1%)
Among test negatives (n = 1139), Site of disease	
Pulmonary	833 (73%)
Extrapulmonary	263 (23%)
Both	37 (3%)
Unknown	6 (1%)
TB treatment outcome (n = 2722)	
Favorable outcome†	1918 (70%)
Unfavorable outcome‡	584 (21%)
Missing	220 (8%)
Among test positives (n = 931), TB treatment outcome	
Favorable outcome†	682 (73%)
Unfavorable outcome‡	162 (17%)
Missing	87 (9%)
Among test negatives (n = 1139), TB treatment outcome	
Favorable outcome†	865 (76%)
Unfavorable outcome‡	197 (17%)
Missing	77 (7%)

* Number of tests exceeds number tested per because each patient could be tested using more than one method.

† Favorable TB treatment outcomes: cure or completion of treatment.

‡ Unfavorable TB treatment outcomes: death, failure or default.

Among all 2722 TB patients, 4% received Xpert testing overall (Table 4). There was a subtle and non-statistically significant increase in proportion of TB treatment cases tested by Xpert: 3.6% in 2012, 4.9% in 2013, and 5.1% in 2014. Of these 118 patients, 64% were positive; 13% of the 76 Xpert-positive cases were resistant to rifampicin, with 2% missing resistance results. Xpert cases were mostly pulmonary in site (70%), with 16% EPTB and 14% both. AFB smear was not performed in 23% (n = 27) of cases where Xpert was done. Among those cases with both tests, there was concordance in 69% (n = 32 positive and 31 negative), Xpert positive and AFB smear negative in 23% (n = 21), and Xpert negative and AFB smear positive in 8% (n = 7).

**Table 4:** Xpert MTB/RIF utilization and outcomes among 2722 adult patients.

	n (%)
Xpert utilization (n = 2722)	
Not done	2602 (96%)
Done	118 (4%)
Missing	2 (<1%)
Among Xpert test done (n = 118), Xpert results	
Positive	76 (64%)
Negative	42 (36%)
Among Xpert test positives (n = 76), Rifampicin resistance	
No	64 (84%)
Yes	10 (13%)
Missing	2 (2%)
Among Xpert test positives (n = 76), Site of disease	
Pulmonary	53 (70%)
Extrapulmonary	12 (16%)
Both	11 (14%)
Among Xpert test positives (n = 76), TB treatment outcome	
Favorable outcome†	61 (80%)
Unfavorable outcome‡	10 (13%)
Missing	5 (7%)
Among Xpert test negatives (n = 42), TB treatment outcome	
Favorable outcome†	32 (76%)
Unfavorable outcome‡	8 (19%)
Missing	2 (5%)

† Favorable TB treatment outcomes: cure or completion of treatment.

‡ Unfavorable TB treatment outcomes: death, failure or default.

Table 5 shows factors associated with an unfavorable TB treatment outcome after adjustment for all other variables in the table. We found no association of unfavorable TB treatment outcomes with age (RR 1.03, 95%CI: 0.96, 1.11) or sex (RR 1.00, 95%CI: 0.86, 1.16). As expected, higher CD4 and currently on ART at TB diagnosis were associated with a reduction in unfavorable TB treatment outcomes, an association that also held in the meta-analysis (S1 Appendix). Lack of documentation of an Xpert test performed had no evidence of an association with an unfavorable TB treatment outcome (RR 1.25, 95%CI: 0.83, 1.90). The Asia-Pacific region had a 60% reduction in the risk and CCASAnet had a 39% increase in the risk of unfavorable TB treatment outcomes for those with Xpert testing compared with the East Africa region (RR 0.40, 95%CI: 0.29–0.57 and 1.39, 1.12–1.72, respectively).

**Table 5:** Multivariable modified Poisson regression analysis of factors associated with an unfavorable TB treatment outcome.

	Relative Risk (95% CI)
Age (per 10 years)	1.03 (0.96, 1.11)
BMI (per 1 kg/m^2^)	0.99 (0.97, 1.01)
CD4 count (200 vs. 50 cells/μL)	0.73 (0.63, 0.85)
CD4 count (500 vs. 50 cells/μL)	0.62 (0.41, 0.92)
Female (vs. Male)	1.00 (0.86, 1.16)
No Xpert (vs. had Xpert)	1.25 (0.83, 1.90)
On ART at TB diagnosis	0.76 (0.64, 0.89)
Region	
East Africa (ref)	
Asia-Pacific	0.40 (0.29, 0.57)
CCASAnet	1.39 (1.12, 1.72)
Central Africa	0.92 (0.74, 1.14)
West Africa	1.15 (0.89, 1.49)

There are 2502 adult patients included in this model; 584 had an unfavorable outcome. 220 patients missing outcomes data were excluded from modeling; among these, 115 (52%) were known transfers. CD4 count is modeled using a restricted cubic spline transformation with 4 knots. If patients on ART are separated by duration of 6+ months or < 6 months, then the adjusted relative risk (ref: No ART) would be: 0.75 (0.60, 0.94) and 0.76 (0.61, 0.95), respectively.

## Discussion

In this large, international study of Xpert MTB/RIF access and utilization, we found that the majority of sites had access to Xpert, but only 4% of TB/HIV co-infected patients in this cohort were tested using Xpert. Eighteen countries participated in this survey and country-specific policy was evolving rapidly during the study period. Additionally, access to Xpert was increasing quickly due to contributions from international non-governmental organizations and public/private partnerships. Therefore, it is difficult to summarize the specific Xpert policy at each site during data collection. However, all data were collected after the WHO guidelines recommending Xpert as the first diagnostic method for suspected HIV/TB co-infected patients. Still, apart from national policy, our results showed a lack of use of Xpert despite availability of the device at the sites. Over half (54%) of the patients with no TB test were treated at facilities with Xpert available on-site. For another 34%, Xpert was available offsite. Thus, for most patients, Xpert was available but it was not used in their case, nor were they tested for TB using any other diagnostic method.

This low utilization is concerning given that this study occurred well over one year after the WHO guidelines recommendation that Xpert be used for TB diagnosis among all TB/HIV co-infected persons. Local study site investigators have hypothesized that there are several possible explanations for the low utilization of Xpert, including cartridge stock-outs, interruptions in electricity, and unreliable transportation of specimens at sites with off-site Xpert testing. Additionally, local study site investigators reported anecdotally that provider education on the WHO guidelines and importance of Xpert utilization may be lacking. WHO recommends that sites routinely monitor the number of cartridges used, shelf-life of cartridges, and estimated delivery time in order to improve the supply-chain management and avoid cartridge stock-outs.[27] High costs, particularly during the start-up period, may impede implementation in low-resource settings, and may include not only the costs of the cartridges and modules but also shipping, calibration kits for maintenance, customs/clearance fees for importation, power supply, staff training, among other costs.[27] Also, implementation is dependent on the speed of enacting revised national TB testing guidelines.

Despite the low utilization, we did not find that lack of Xpert testing was associated with an unfavorable TB treatment outcome. Overall, 70% of the patients in our study had a favorable outcome. While all patients were diagnosed as co-infected with HIV/TB and initiated TB treatment, nearly one-quarter of patients (24%) had no record of any TB test, showing a heavy reliance upon empiric TB treatment. Other studies in low-resource settings have shown a similar reliance upon empiric treatment [28–30] and have suggested that this may reduce the population-level impact of Xpert if it displaces true-positive empiric treatment.[31] Placement of the Xpert machine likely influences the choice between initiating immediate empiric treatment versus waiting for diagnostic tests, as a two-hour wait for a point-of-care result may be preferential to empiric treatment, but this would not be the case if samples are sent away to an off-site lab. Additionally, off-site Xpert placement may require greater transport burden for patients or the need to transport specimen when cold-chain infrastructure is not available. In our study, only 13% of sites had Xpert based within the clinic, and it was co-located within 20% of the facilities. Thus, for the remaining 67%, on-site use was not available, likely leading to the high frequency of empiric treatment we observed.

This study emphasizes that operational research must address global implementation challenges of Xpert in order to maximize the potential benefits of this new technology. In contrast to two recent multi-country Xpert studies, our study did not provide Xpert machines to sites [18] or a funding mechanism to procure machines.[16] Additionally, we also surveyed the sites directly, rather than relying on national program representatives who were the sources of information in another study.[19] As such, we are able to report on the field implementation of Xpert as reported by participating sites.

While Xpert was used only among a small proportion of cases, 64% were Xpert-positive. A Cochrane review reported a pooled sensitivity of Xpert as 79% (95% CI 70–86%) among people living with HIV. However, this pooled sensitivity differed depending on smear status with 61% (95% CI 40–81%) among smear-negative persons compared to 97% (95% CI 90–99%) among smear-positive persons.[10] In our study, there was a mix of Xpert use in the absence of smear (n = 27) and in conjunction with smear (n = 91). Among those with negative smears (n = 52), 40% were positive by Xpert and among those with positive smears (n = 39), 82% were positive by Xpert. Thus, the Xpert positivity difference we observed based on smear results is consistent with that of other studies.

Our prevalence of rifampicin resistance, a marker of MDR-TB, was 13%. In 2015, the WHO estimated that 3.9% (95% CI 2.7–5.1%) of new cases and 21% (95% CI 15–28%) of previously treated cases had MDR- or rifampicin resistant-TB [1]. Given the prevalence of HIV-associated MDR-TB is similar to the prevalence of MDR-TB in the general population,[32] these findings suggest that Xpert testing may have been reserved for persons suspected of having MDR-TB, rather than being used routinely for diagnosis among all HIV-infected persons. This runs counter to the WHO guidelines recommending Xpert as the first-line TB test for all patients suspected of HIV/TB co-infection.

Among 30% of the TB cases diagnosed with Xpert, the site of disease was extrapulmonary or both pulmonary and EPTB. Thus our findings demonstrate the utility of Xpert for testing non-sputum samples and again suggest that Xpert is being utilized for testing of non-sputum samples as recommended by the WHO in a recent update on Xpert policy.[14]

We acknowledge several limitations related to this study. First, due to resource constraints, data for all HIV/TB cases could not be collected and the patient population included varied by site. This introduces the possibility of selection bias, although if sites were unable to enter data for all TB cases they were asked to select cases at random. Second, the retrospective nature of our data collection may also result in selection bias or information bias. Related to retrospective data, a possible lag in case identification and/or data entry may have contributed to fewer cases included in 2014. Third, it is possible that Xpert use has increased since our site-level data were collected in 2012, but our results represent a time period well over a year after the WHO guidelines announcement. Additionally, it is unlikely that sites lost access to Xpert over the study period and an increase in access to Xpert at the site-level would further strengthen our conclusion that Xpert utilization was low despite access to the technology. Fourth, data for the timing of TB testing to TB treatment initiation were not available and we were, therefore, unable to measure and control for the use of empiric anti-TB therapy. Similarly, data for the timing of TB treatment outcomes were not available so that information from patients who were lost or transferred could be included in a censored time-to-event analysis.

In conclusion, our data suggest that in the low- and middle-income countries participating in this study, implementation of Xpert is low. In order to fulfill the promise of Xpert as a ground-breaking TB diagnostic, we must ensure that its implementation meets the WHO guidelines. Our findings show that there is a long way yet to go in achieving widespread clinic-level access to Xpert and patient-level implementation. Future study should focus on the barriers to implementation of Xpert use in resource-limited settings.

## Supporting information

S1 Appendix Modified Poisson Regression Stratified by Region and Combined using Meta-Analysis: factors associated with an unfavorable TB treatment outcome. The combined relative risks (RRs) and 95% CIs were computed based on the results of the region-specific RRs using the meta- analysis approach of DerSimonian and Laird, a random effects method which makes no assumption regarding homogeneity across sites.
(PDF)

S2 Appendix Membership of the International Epidemiologic Databases to Evaluate AIDS (IeDEA) collaboration for participating. (PDF)
