# Supplementary material for: Low implementation of Xpert MTB/RIF among HIV/TB co-infected adults in the International epidemiologic Databases to Evaluate AIDS (IeDEA) program
Source: PLoS One. 2017 Feb 9;12(2):e0171384. doi: 10.1371/journal.pone.0171384 (PMC5300213; doi:10.1371/journal.pone.0171384)
Supplement: S1 Appendix — The combined relative risks (RRs) and 95% CIs were computed based on the results of the region-specific RRs using the meta- analysis approach of DerSimonian and Laird, a random effects method which makes no assumption regarding homogeneity across sites. (PDF) [file pone.0171384.s001.pdf]

S1 Appendix

S1 Table. Modified Poisson Regression Stratified by Region and Combined using Meta-Analysis: factors associated with an unfavorable TB treatment outcome

	Asia-Pacific<br>Relative Risk (95% CI)	CCASAnet<br>Relative Risk (95% CI)	Central Africa<br>Relative Risk (95% CI)
Age (per 10 years)	1.11 (0.70, 1.77)	1.02 (0.86, 1.22)	0.97 (0.78, 1.20)
BMI (per 1 unit)	0.88 (0.75, 1.03)	1.03 (0.98, 1.09)	1.00 (0.95, 1.04)
CD4 (200 vs. 50 cells)	0.69 (0.43, 1.12)	1.02 (0.45, 2.27)	0.86 (0.70, 1.05)
CD4 (500 vs. 50 cells)	0.33 (0.08, 1.39)	1.20 (0.05, 27.33)	0.63 (0.34, 1.16)
Female (vs. Male)	1.80 (0.83, 3.88)	1.48 (1.02, 2.15)	0.97 (0.64, 1.45)
No Xpert (vs. had Xpert)	1.96 (0.48, 8.02)	0.96 (0.52, 1.77)	1.89 (0.33, 10.78)
On ART at TB diagnosis	0.81 (0.41, 1.59)	1.22 (0.84, 1.79)	0.63 (0.41, 0.96)
	East Africa<br>Relative Risk (95% CI)	Western Africa<br>Relative Risk (95% CI)	Combined<br>Relative Risk (95% CI)
Age (per 10 years)	1.03 (0.94, 1.14)	0.95 (0.72, 1.27)	1.02 (0.95, 1.10)
BMI (per 1 unit)	0.99 (0.96, 1.01)	0.98 (0.92, 1.04)	0.99 (0.97, 1.02)
CD4 (200 vs. 50 cells)	0.66 (0.54, 0.81)	0.80 (0.54, 1.18)	0.76 (0.67, 0.86)
CD4 (500 vs. 50 cells)	0.43 (0.18, 1.01)	0.50 (0.16, 1.63)	0.53 (0.34, 0.81)
Female (vs. Male)	0.92 (0.76, 1.11)	0.91 (0.55, 1.50)	1.08 (0.86, 1.37)
No Xpert (vs. had Xpert)	1.17 (0.57, 2.38)	2.01 (0.52, 7.74)	1.21 (0.80, 1.81)
On ART at TB diagnosis	0.70 (0.56, 0.88)	0.71 (0.42, 1.20)	0.79 (0.62, 1.01)

The combined relative risks (RRs) and 95% CIs were computed based on the results of the region-specific RRs using the meta- analysis approach of DerSimonian and Laird, a random effects method which makes no assumption regarding homogeneity across sites.
